# Diethyl 4-oxo-4*H*-[1,4′-bi­quinoline]-3,3′-di­carboxyl­ate

**DOI:** 10.1107/S1600536814007314

**Published:** 2014-04-05

**Authors:** Yoshinobu Ishikawa, Nanako Yoshida

**Affiliations:** a School of Pharmaceutical Sciences, University of Shizuoka, 52-1 Yada, Suruga-ku, Shizuoka 422-8526, Japan

## Abstract

In the title mol­ecule, C_24_H_20_N_2_O_5_, the quinoline and quinolinone moieties are practically perpendicular to each other, forming a dihedral angle of 89.06 (3)°. In the crystal, each moiety forms coplanar π-stacked couples with the respective inversion equivalents. The quinolinone moieties overlap with their benzene rings with a centroid–centroid separation of 3.641 (2) Å, whereas the quinoline moieties overlap with their pyridine rings with a separation of 3.592 (2) Å. The resulting supra­molecular chains propargate along [101].

## Related literature   

For the background to this study, see: Ishikawa & Fujii (2011 ▶). For a related compound, see: Kajihara (1965 ▶).
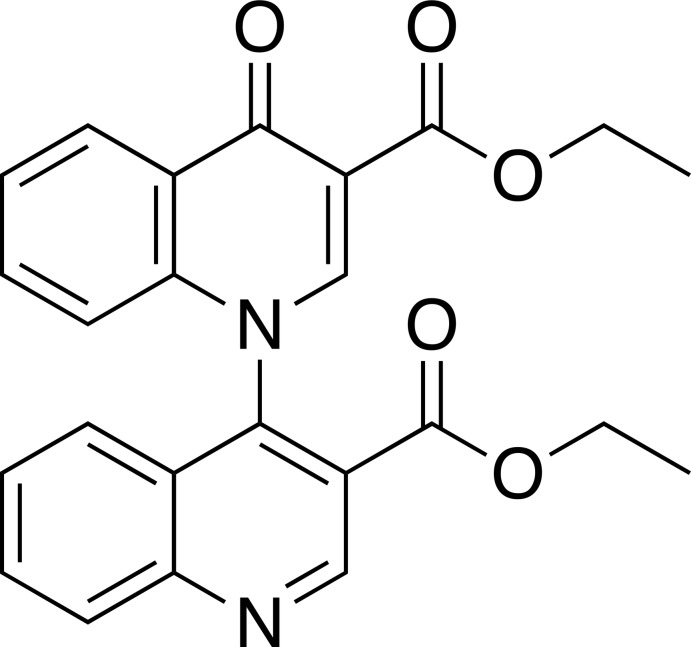


## Experimental   

### 

#### Crystal data  

C_24_H_20_N_2_O_5_
*M* *_r_* = 416.43
Triclinic, 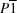

*a* = 7.4478 (16) Å
*b* = 11.284 (5) Å
*c* = 12.583 (5) Å
α = 107.78 (3)°
β = 92.96 (3)°
γ = 102.85 (3)°
*V* = 973.5 (6) Å^3^
*Z* = 2
Mo *K*α radiation
μ = 0.10 mm^−1^
*T* = 100 K
0.25 × 0.20 × 0.15 mm

#### Data collection  

Rigaku AFC-7R diffractometer
5486 measured reflections
4479 independent reflections
3077 reflections with *F* ^2^ > 2σ(*F* ^2^)
*R*_int_ = 0.034
3 standard reflections every 150 reflections intensity decay: −0.2%

#### Refinement  

*R*[*F* ^2^ > 2σ(*F* ^2^)] = 0.052
*wR*(*F* ^2^) = 0.159
*S* = 1.02
4479 reflections
282 parameters
H-atom parameters constrained
Δρ_max_ = 0.26 e Å^−3^
Δρ_min_ = −0.31 e Å^−3^

### 

Data collection: *WinAFC Diffractometer Control Software* (Rigaku, 1999 ▶); cell refinement: *WinAFC Diffractometer Control Software*; data reduction: *WinAFC Diffractometer Control Software*; program(s) used to solve structure: *SIR2008* (Burla *et al.*, 2007 ▶); program(s) used to refine structure: *SHELXL97* (Sheldrick, 2008 ▶); molecular graphics: *CrystalStructure* (Rigaku, 2010 ▶); software used to prepare material for publication: *CrystalStructure*.

## Supplementary Material

Crystal structure: contains datablock(s) General, I. DOI: 10.1107/S1600536814007314/ld2124sup1.cif

Structure factors: contains datablock(s) I. DOI: 10.1107/S1600536814007314/ld2124Isup2.hkl

Supporting information file. DOI: 10.1107/S1600536814007314/ld2124Isup3.cml

CCDC reference: 994976

Additional supporting information:  crystallographic information; 3D view; checkCIF report

## Figures and Tables

**Fig. 1.**
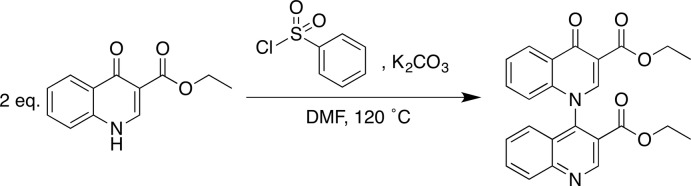
Reaction scheme for the title compound.

**Fig. 2.**
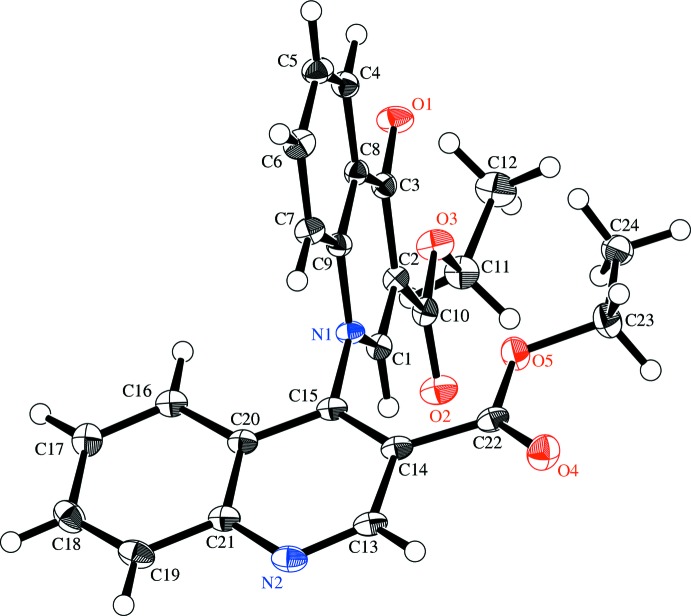
The molecular structure of the title compound, with displacement ellipsoids drawn at the 50% probability level. Hydrogen atoms are shown as small spheres of arbitrary radius.

**Fig. 3.**
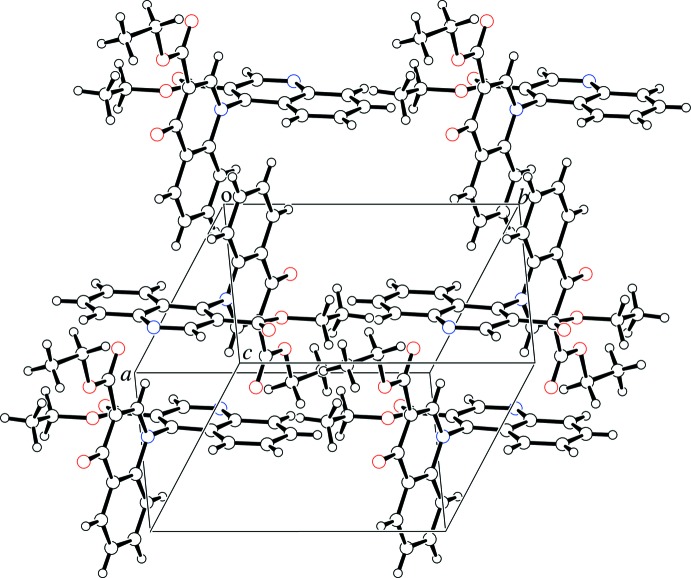
A crystal packing view of the title compound.

## supplementary crystallographic information

### 1. Comment

4-Quinolones show inhibition not only to Gram-negative and Gram-positive bacteria, but also to human immunodeficiency virus (HIV). The inhibition to HIV is derived from their chelating ability to metal ions in the active site of the metalloenzyme HIV integrase. According to our inhibitor design targeting the metalloenzyme influenza virus RNA polymerase (Ishikawa & Fujii, 2011), we tried to synthesize a 4-quinolone derivative bearing a benzensulfonyl group. Reaction of ethyl 4-oxo-1,4-dihydroquinoline-3-carboxylate with benzenesulfonyl chloride in the presence of K_2_CO_3_ in *N*,*N*-dimethylformamide (DMF) at 120 °C gave the white solid after purification by silica gel chromatography (Fig.1).

The crystallographic analysis revealed that it is a self-condensation product of ethyl 4-oxo-1,4-dihydroquinoline-3-carboxylate with a loss of one water molecule, as shown in Fig.2. This structure well accounts for the ^1^H NMR and MS spectra. The C–N bond formation should occur *via* the formation of a benzenesulfonate intermediate. The quinoline rings are approximately perpendicular to each other [dihedral angle = 90.94 (3)°]. In the crystal, the molecule is assembled *via* stacking interaction with its inversion equivalents^i,ii^ [centroid-centroid distances between the benzene rings of the upper quinoline units = 3.641 (2) Å (i: -*x*, -*y*, -*z*) and between the pyridine rings of the lower quinoline units = 3.592 (2) Å (ii: -*x* + 1, -*y*, -*z* + 1)]. As a result, the molecules form chains along [101] direction, as shown in Fig.3. Report on the synthesis of 1,4'-quinolines is scarce (Kajihara, 1965).

### 2. Experimental

In a Schlenk tube under nitrogen atmosphere, the mixture of ethyl 4-oxo-1,4-dihydroquinoline-3-carboxylate (0.20 g, 1.0 mmol), benzenesufonyl chloride (0.35 g, 2.0 mmol), K_2_CO_3_ (0.28 g, 2.0 mmol) in 5 ml of DMF were stirred at 120 °C overnight. After cooling to room temperature water was added, and the mixture was extracted with ethyl acetate three times. The extract was dried over anhydrous Na_2_SO_4_, and purified by column chromatography on silica gel (ethyl acetate: ethanol = 180: 5, yield: 33%). ^1^H NMR (400 MHz, DMSO-*d*_6_): *δ* = 0.87 (t, 3H, *J* = 7.1 Hz), 1.24 (t, 3H, *J* = 7.1 Hz), 4.07 (m, 2H), 4.21 (q, 2H, *J* = 7.1 Hz), 6.71 (d, 1H, *J* = 7.3 Hz), 7.50 (m, 2H), 7.60 (d, 1H, *J* = 8.3 Hz), 7.74 (t, 1H, *J* = 7.6 Hz), 8.05 (t, 1H, *J* = 7.8 Hz), 8.33 (d, 1H, *J* = 8.3 Hz), 8.34 (d, 1H, *J* = 8.3 Hz), 8.74 (s, 1H), 9.57 (s, 1H). DART-MS calcd for [C_24_H_20_N_2_O_5_ + H^+^]: 417.188, found 417.150. Single crystals suitable for X-ray diffraction were obtained by slow evapolation of an ethyl acetate solution of the title compound at room temperature.

### 3. Refinement

The C(*sp*^2^)-bound [C–H 0.95 Å, *U*_iso_(H) = 1.2*U*_eq_(C)] and methylene [C–H 0.99 Å, *U*_iso_(H) = 1.2*U*_eq_(C)] hydrogen atoms were placed in geometrical positions and refined using a riding model. A rotating group model was applied to the methyl groups with distance constraint [C–H = 0.98 Å, *U*_iso_(H) = 1.2*U*_eq_(C)].

### Crystal data

**Table:**

C_24_H_20_N_2_O_5_	*Z* = 2
*M**_r_* = 416.43	*F*(000) = 436.00
Triclinic, *P*1	*D*_x_ = 1.421 Mg m^−^^3^
Hall symbol: -P 1	Mo *K*α radiation, λ = 0.71069 Å
*a* = 7.4478 (16) Å	Cell parameters from 25 reflections
*b* = 11.284 (5) Å	θ = 15.3–17.3°
*c* = 12.583 (5) Å	µ = 0.10 mm^−^^1^
α = 107.78 (3)°	*T* = 100 K
β = 92.96 (3)°	Plate, colorless
γ = 102.85 (3)°	0.25 × 0.20 × 0.15 mm
*V* = 973.5 (6) Å^3^	

### Data collection

**Table:**

Rigaku AFC-7R diffractometer	θ_max_ = 27.5°
ω–2θ scans	*h* = −9→5
5486 measured reflections	*k* = −14→14
4479 independent reflections	*l* = −16→16
3077 reflections with *F*^2^ > 2σ(*F*^2^)	3 standard reflections every 150 reflections
*R*_int_ = 0.034	intensity decay: −0.2%

### Refinement

**Table:**

Refinement on *F*^2^	Secondary atom site location: difference Fourier map
*R*[*F*^2^ > 2σ(*F*^2^)] = 0.052	Hydrogen site location: inferred from neighbouring sites
*wR*(*F*^2^) = 0.159	H-atom parameters constrained
*S* = 1.02	*w* = 1/[σ^2^(*F*_o_^2^) + (0.0934*P*)^2^ + 0.0738*P*] where *P* = (*F*_o_^2^ + 2*F*_c_^2^)/3
4479 reflections	(Δ/σ)_max_ < 0.001
282 parameters	Δρ_max_ = 0.26 e Å^−^^3^
0 restraints	Δρ_min_ = −0.31 e Å^−^^3^
Primary atom site location: structure-invariant direct methods	

### Special details

**Table:**

Refinement. Refinement was performed using all reflections. The weighted *R*-factor (*wR*) and goodness of fit (*S*) are based on *F*^2^. *R*-factor (gt) are based on *F*. The threshold expression of *F*^2^ > 2.0 σ(*F*^2^) is used only for calculating *R*-factor (gt).

### Fractional atomic coordinates and isotropic or equivalent isotropic displacement parameters (Å^2^)

**Table:**

	*x*	*y*	*z*	*U*_iso_*/*U*_eq_	
O1	0.3332 (2)	0.32182 (13)	0.08577 (12)	0.0232 (4)	
O2	0.80212 (19)	0.33534 (14)	0.30167 (12)	0.0236 (4)	
O3	0.69998 (19)	0.40856 (13)	0.16873 (12)	0.0222 (4)	
O4	0.1676 (2)	0.17036 (14)	0.61313 (12)	0.0259 (4)	
O5	0.2323 (2)	0.24073 (13)	0.46750 (12)	0.0228 (4)	
N1	0.3020 (2)	0.08516 (14)	0.27797 (13)	0.0145 (4)	
N2	0.2925 (3)	−0.17369 (16)	0.46462 (14)	0.0206 (4)	
C1	0.4640 (3)	0.16992 (17)	0.28111 (15)	0.0149 (4)	
C2	0.4860 (3)	0.25268 (17)	0.21964 (15)	0.0149 (4)	
C3	0.3287 (3)	0.25386 (17)	0.14649 (15)	0.0150 (4)	
C4	−0.0110 (3)	0.15898 (18)	0.08414 (16)	0.0177 (4)	
C5	−0.1760 (3)	0.07462 (19)	0.08318 (16)	0.0203 (4)	
C6	−0.1832 (3)	−0.00914 (19)	0.14607 (16)	0.0202 (5)	
C7	−0.0263 (3)	−0.00679 (18)	0.21064 (16)	0.0170 (4)	
C8	0.1514 (3)	0.16402 (17)	0.14924 (15)	0.0145 (4)	
C9	0.1409 (3)	0.08087 (17)	0.21275 (15)	0.0142 (4)	
C10	0.6784 (3)	0.33482 (17)	0.23514 (16)	0.0159 (4)	
C11	0.8869 (3)	0.48947 (19)	0.17788 (18)	0.0214 (5)	
C12	0.8689 (3)	0.5903 (2)	0.1253 (2)	0.0280 (5)	
C13	0.2596 (3)	−0.06055 (19)	0.50924 (17)	0.0185 (4)	
C14	0.2587 (3)	0.03214 (18)	0.45333 (16)	0.0165 (4)	
C15	0.2959 (3)	−0.00050 (17)	0.34353 (16)	0.0155 (4)	
C16	0.3614 (3)	−0.16464 (18)	0.17583 (16)	0.0174 (4)	
C17	0.3957 (3)	−0.28265 (19)	0.12941 (17)	0.0209 (5)	
C18	0.3959 (3)	−0.36552 (19)	0.19449 (18)	0.0231 (5)	
C19	0.3611 (3)	−0.32810 (19)	0.30362 (18)	0.0222 (5)	
C20	0.3277 (3)	−0.12283 (17)	0.28961 (16)	0.0159 (4)	
C21	0.3266 (3)	−0.20668 (18)	0.35423 (16)	0.0176 (4)	
C22	0.2150 (3)	0.15395 (18)	0.52060 (16)	0.0174 (4)	
C23	0.1895 (3)	0.36186 (19)	0.52667 (18)	0.0233 (5)	
C24	0.2266 (3)	0.44432 (19)	0.45232 (18)	0.0233 (5)	
H1	0.5688	0.1723	0.3288	0.0179*	
H2	−0.0066	0.2145	0.0403	0.0212*	
H3	−0.2852	0.0731	0.0398	0.0244*	
H4	−0.2973	−0.0681	0.1442	0.0242*	
H5	−0.0315	−0.0638	0.2531	0.0203*	
H6	0.2342	−0.0384	0.5851	0.0222*	
H7	0.3602	−0.1101	0.1314	0.0209*	
H8	0.4194	−0.3092	0.0534	0.0251*	
H9	0.4203	−0.4472	0.1620	0.0277*	
H10	0.3601	−0.3847	0.3462	0.0266*	
H11A	0.9685	0.4372	0.1379	0.0257*	
H12B	0.9410	0.5304	0.2579	0.0257*	
H13A	0.7940	0.6446	0.1684	0.0336*	
H14B	0.8083	0.5484	0.0476	0.0336*	
H15C	0.9925	0.6435	0.1257	0.0336*	
H16A	0.2689	0.4041	0.6001	0.0280*	
H17B	0.0578	0.3469	0.5402	0.0280*	
H18A	0.1391	0.4052	0.3829	0.0279*	
H19B	0.3539	0.4514	0.4338	0.0279*	
H20C	0.2109	0.5301	0.4918	0.0279*	

### Atomic displacement parameters (Å^2^)

**Table:**

	*U*^11^	*U*^22^	*U*^33^	*U*^12^	*U*^13^	*U*^23^
O1	0.0248 (8)	0.0220 (8)	0.0254 (8)	0.0006 (6)	−0.0014 (6)	0.0159 (6)
O2	0.0185 (7)	0.0263 (8)	0.0259 (8)	−0.0008 (6)	−0.0013 (6)	0.0135 (7)
O3	0.0173 (7)	0.0230 (8)	0.0266 (8)	−0.0041 (6)	0.0017 (6)	0.0151 (7)
O4	0.0323 (9)	0.0269 (8)	0.0198 (8)	0.0061 (7)	0.0080 (7)	0.0095 (7)
O5	0.0333 (9)	0.0193 (7)	0.0195 (8)	0.0094 (7)	0.0091 (6)	0.0088 (6)
N1	0.0132 (8)	0.0161 (8)	0.0155 (8)	0.0018 (6)	0.0009 (6)	0.0085 (7)
N2	0.0180 (9)	0.0231 (9)	0.0247 (9)	0.0027 (7)	0.0017 (7)	0.0151 (8)
C1	0.0135 (9)	0.0149 (9)	0.0156 (9)	0.0023 (7)	0.0022 (7)	0.0048 (8)
C2	0.0142 (9)	0.0157 (9)	0.0143 (9)	0.0024 (7)	0.0019 (7)	0.0049 (7)
C3	0.0163 (9)	0.0129 (9)	0.0151 (9)	0.0021 (7)	0.0015 (7)	0.0049 (7)
C4	0.0200 (10)	0.0177 (9)	0.0171 (9)	0.0064 (8)	0.0029 (8)	0.0070 (8)
C5	0.0158 (9)	0.0265 (11)	0.0192 (10)	0.0070 (8)	0.0008 (8)	0.0073 (9)
C6	0.0147 (9)	0.0241 (11)	0.0209 (10)	0.0020 (8)	0.0029 (8)	0.0079 (9)
C7	0.0163 (9)	0.0190 (10)	0.0169 (9)	0.0019 (8)	0.0040 (7)	0.0093 (8)
C8	0.0153 (9)	0.0146 (9)	0.0134 (9)	0.0038 (8)	0.0024 (7)	0.0042 (7)
C9	0.0135 (9)	0.0157 (9)	0.0142 (9)	0.0044 (7)	0.0017 (7)	0.0053 (8)
C10	0.0168 (9)	0.0139 (9)	0.0158 (9)	0.0007 (7)	0.0037 (7)	0.0051 (7)
C11	0.0155 (10)	0.0217 (10)	0.0268 (11)	−0.0013 (8)	0.0047 (8)	0.0116 (9)
C12	0.0221 (11)	0.0270 (12)	0.0379 (13)	0.0001 (9)	0.0045 (10)	0.0189 (10)
C13	0.0149 (9)	0.0248 (10)	0.0180 (9)	0.0030 (8)	0.0008 (8)	0.0119 (8)
C14	0.0123 (9)	0.0199 (10)	0.0179 (10)	0.0004 (7)	−0.0002 (7)	0.0100 (8)
C15	0.0120 (9)	0.0171 (9)	0.0180 (9)	−0.0001 (7)	−0.0006 (7)	0.0098 (8)
C16	0.0130 (9)	0.0194 (10)	0.0206 (10)	0.0006 (8)	0.0019 (7)	0.0102 (8)
C17	0.0166 (10)	0.0206 (10)	0.0234 (10)	0.0015 (8)	0.0040 (8)	0.0062 (8)
C18	0.0182 (10)	0.0176 (10)	0.0332 (12)	0.0040 (8)	0.0026 (9)	0.0083 (9)
C19	0.0183 (10)	0.0205 (10)	0.0319 (12)	0.0029 (8)	0.0024 (9)	0.0159 (9)
C20	0.0109 (9)	0.0165 (9)	0.0199 (10)	0.0001 (7)	−0.0005 (7)	0.0083 (8)
C21	0.0137 (9)	0.0178 (10)	0.0216 (10)	0.0000 (8)	−0.0004 (8)	0.0101 (8)
C22	0.0131 (9)	0.0220 (10)	0.0167 (9)	0.0001 (8)	−0.0003 (7)	0.0092 (8)
C23	0.0271 (11)	0.0204 (10)	0.0243 (11)	0.0090 (9)	0.0090 (9)	0.0068 (9)
C24	0.0229 (11)	0.0223 (11)	0.0252 (11)	0.0062 (9)	0.0014 (9)	0.0085 (9)

### Geometric parameters (Å, º)

**Table:**

O1—C3	1.234 (3)	C16—C17	1.368 (3)
O2—C10	1.210 (3)	C16—C20	1.419 (3)
O3—C10	1.340 (3)	C17—C18	1.419 (4)
O3—C11	1.462 (3)	C18—C19	1.365 (4)
O4—C22	1.206 (3)	C19—C21	1.410 (3)
O5—C22	1.332 (3)	C20—C21	1.422 (4)
O5—C23	1.456 (3)	C23—C24	1.504 (4)
N1—C1	1.353 (3)	C1—H1	0.950
N1—C9	1.402 (3)	C4—H2	0.950
N1—C15	1.445 (3)	C5—H3	0.950
N2—C13	1.312 (3)	C6—H4	0.950
N2—C21	1.377 (3)	C7—H5	0.950
C1—C2	1.372 (3)	C11—H11A	0.990
C2—C3	1.457 (3)	C11—H12B	0.990
C2—C10	1.490 (3)	C12—H13A	0.980
C3—C8	1.484 (3)	C12—H14B	0.980
C4—C5	1.374 (3)	C12—H15C	0.980
C4—C8	1.405 (3)	C13—H6	0.950
C5—C6	1.400 (4)	C16—H7	0.950
C6—C7	1.379 (3)	C17—H8	0.950
C7—C9	1.401 (3)	C18—H9	0.950
C8—C9	1.399 (3)	C19—H10	0.950
C11—C12	1.506 (4)	C23—H16A	0.990
C13—C14	1.429 (4)	C23—H17B	0.990
C14—C15	1.377 (3)	C24—H18A	0.980
C14—C22	1.493 (3)	C24—H19B	0.980
C15—C20	1.418 (3)	C24—H20C	0.980
			
O1···O3	2.721 (2)	C16···H3^ix^	3.2503
O1···C4	2.797 (3)	C16···H4^iv^	2.6347
O1···C10	3.054 (3)	C16···H15C^xii^	2.9958
O2···C1	2.728 (3)	C17···H4^iv^	2.8881
O2···C11	2.668 (4)	C17···H13A^x^	3.3009
O3···C1	3.580 (3)	C17···H14B^ii^	3.1314
O3···C3	2.872 (3)	C17···H15C^xii^	2.9258
O4···C13	2.788 (3)	C18···H11A^xii^	3.3525
O4···C23	2.679 (4)	C18···H12B^xii^	3.5459
O5···N1	2.649 (3)	C18···H13A^x^	2.9809
O5···C1	3.000 (3)	C18···H14B^ii^	3.1837
O5···C9	3.113 (3)	C18···H15C^xii^	3.1156
O5···C15	2.850 (3)	C19···H11A^xii^	3.5303
N1···C3	2.862 (3)	C19···H12B^xii^	3.1146
N1···C16	2.862 (3)	C19···H15C^xii^	3.3449
N1···C22	3.053 (3)	C19···H16A^vii^	3.3374
N2···C15	2.818 (4)	C19···H18A^x^	3.5463
C1···C8	2.765 (3)	C19···H19B^x^	3.3639
C1···C14	3.285 (4)	C19···H20C^x^	3.3479
C1···C16	3.485 (3)	C20···H4^iv^	3.4608
C1···C20	3.269 (3)	C20···H6^vii^	3.3943
C2···C9	2.834 (3)	C20···H15C^xii^	3.2647
C4···C7	2.791 (4)	C21···H12B^xii^	3.4887
C5···C9	2.773 (3)	C21···H15C^xii^	3.4265
C6···C8	2.794 (3)	C21···H17B^vi^	3.4823
C7···C14	3.482 (3)	C22···H5^vi^	3.5442
C7···C15	2.828 (3)	C22···H6^vi^	3.3471
C7···C20	3.422 (4)	C22···H10^vii^	3.5672
C9···C14	3.346 (4)	C23···H10^vii^	3.5622
C9···C16	3.450 (4)	C23···H12B^v^	2.9250
C9···C20	3.286 (4)	C23···H19B^v^	3.5003
C13···C19	3.581 (4)	C23···H20C^xiii^	3.4856
C13···C20	2.740 (3)	C24···H10^iii^	2.7110
C14···C21	2.773 (3)	C24···H17B^xiii^	3.4851
C16···C19	2.792 (4)	C24···H19B^v^	3.1740
C17···C21	2.805 (3)	C24···H20C^xiii^	3.4241
C18···C20	2.805 (3)	H1···N2^vii^	2.7418
O1···C12^i^	3.487 (4)	H1···C6^iv^	3.4832
O1···C17^ii^	3.440 (3)	H1···C13^vii^	3.0655
O1···C18^iii^	3.285 (3)	H1···H4^iv^	3.3676
O2···C4^iv^	3.419 (3)	H1···H6^vii^	2.7266
O2···C5^iv^	3.400 (3)	H1···H20C^v^	3.4058
O2···C23^v^	3.424 (3)	H2···O3^viii^	3.5373
O2···C24^iv^	3.366 (3)	H2···C6^ix^	3.3689
O2···C24^v^	3.378 (3)	H2···C7^ix^	3.3674
O3···C17^ii^	3.552 (3)	H2···C11^viii^	3.3478
O4···N2^vi^	3.526 (3)	H2···C12^i^	3.5014
O4···C7^vi^	3.363 (3)	H2···C16^ix^	3.5155
O4···C13^vi^	3.256 (3)	H2···H7^ix^	3.0777
O4···C14^vi^	3.366 (3)	H2···H11A^viii^	2.4907
O4···C19^vii^	3.506 (3)	H2···H14B^i^	3.2824
N2···O4^vi^	3.526 (3)	H2···H15C^i^	2.9882
N2···C1^vii^	3.579 (3)	H3···C2^viii^	3.3558
N2···C14^vii^	3.324 (3)	H3···C8^ix^	3.3698
N2···C15^vii^	3.570 (3)	H3···C9^ix^	3.4764
C1···N2^vii^	3.579 (3)	H3···C10^viii^	3.2903
C4···O2^viii^	3.419 (3)	H3···C16^ix^	3.2503
C4···C5^ix^	3.444 (3)	H3···H3^xiv^	3.2050
C4···C6^ix^	3.445 (3)	H3···H7^viii^	3.4521
C5···O2^viii^	3.400 (3)	H3···H7^ix^	2.3788
C5···C4^ix^	3.444 (3)	H3···H8^ix^	3.5271
C5···C8^ix^	3.362 (3)	H4···O1^ix^	3.3409
C5···C10^viii^	3.414 (3)	H4···C1^viii^	3.5997
C6···C4^ix^	3.445 (3)	H4···C3^ix^	3.5833
C6···C16^viii^	3.550 (3)	H4···C16^viii^	2.6347
C7···O4^vi^	3.363 (3)	H4···C17^viii^	2.8881
C8···C5^ix^	3.362 (3)	H4···C20^viii^	3.4608
C10···C5^iv^	3.414 (3)	H4···H1^viii^	3.3676
C12···O1^i^	3.487 (4)	H4···H6^vi^	3.2171
C13···O4^vi^	3.256 (3)	H4···H7^viii^	2.4759
C13···C13^vii^	3.585 (3)	H4···H8^viii^	2.9084
C13···C14^vii^	3.524 (3)	H4···H13A^xii^	3.5528
C13···C15^vii^	3.527 (3)	H5···O4^vi^	2.4848
C13···C22^vi^	3.422 (3)	H5···C13^vi^	3.5802
C14···O4^vi^	3.366 (3)	H5···C22^vi^	3.5442
C14···N2^vii^	3.324 (3)	H5···H6^vi^	2.7225
C14···C13^vii^	3.524 (3)	H5···H13A^xii^	3.0760
C14···C21^vii^	3.591 (3)	H5···H15C^xii^	3.2508
C15···N2^vii^	3.570 (3)	H6···O4^vi^	3.5171
C15···C13^vii^	3.527 (3)	H6···C1^vii^	3.5653
C16···C6^iv^	3.550 (3)	H6···C6^vi^	3.3152
C17···O1^ii^	3.440 (3)	H6···C7^vi^	3.0373
C17···O3^ii^	3.552 (3)	H6···C15^vii^	3.4641
C18···O1^x^	3.285 (3)	H6···C20^vii^	3.3943
C19···O4^vii^	3.506 (3)	H6···C22^vi^	3.3471
C19···C22^vii^	3.548 (3)	H6···H1^vii^	2.7266
C21···C14^vii^	3.591 (3)	H6···H4^vi^	3.2171
C21···C22^vii^	3.530 (3)	H6···H5^vi^	2.7225
C22···C13^vi^	3.422 (3)	H6···H17B^vi^	3.5016
C22···C19^vii^	3.548 (3)	H7···C4^ix^	3.4854
C22···C21^vii^	3.530 (3)	H7···C5^ix^	3.1398
C23···O2^v^	3.424 (3)	H7···C6^iv^	3.3133
C24···O2^viii^	3.366 (3)	H7···H2^ix^	3.0777
C24···O2^v^	3.378 (3)	H7···H3^iv^	3.4521
O1···H2	2.5024	H7···H3^ix^	2.3788
O2···H1	2.3541	H7···H4^iv^	2.4759
O2···H11A	2.8600	H7···H15C^xii^	3.4211
O2···H12B	2.4509	H8···O1^ii^	2.6034
O3···H13A	2.6002	H8···O3^ii^	2.6912
O3···H14B	2.5486	H8···C3^ii^	3.3404
O3···H15C	3.2399	H8···C10^ii^	3.5754
O4···H6	2.4392	H8···C11^ii^	3.3825
O4···H16A	2.6354	H8···C12^ii^	3.4407
O4···H17B	2.6916	H8···H3^ix^	3.5271
O5···H1	3.2272	H8···H4^iv^	2.9084
O5···H18A	2.6002	H8···H11A^ii^	3.3950
O5···H19B	2.5194	H8···H13A^x^	3.2930
O5···H20C	3.2261	H8···H14B^ii^	2.7242
N1···H5	2.6148	H8···H15C^xii^	3.3256
N1···H7	2.5433	H9···O1^x^	2.4059
N2···H10	2.5667	H9···O3^x^	2.9268
C1···H7	3.0427	H9···C3^x^	3.2308
C1···H19B	3.4704	H9···C12^x^	3.3470
C2···H19B	3.2896	H9···H11A^xii^	3.2888
C3···H1	3.3018	H9···H13A^x^	2.7331
C3···H2	2.6493	H9···H14B^x^	3.2979
C3···H18A	3.4876	H9···H14B^ii^	2.8269
C3···H19B	3.5909	H9···H16A^vii^	3.5282
C4···H4	3.2566	H10···O5^vii^	3.4783
C5···H5	3.2740	H10···C22^vii^	3.5672
C6···H2	3.2599	H10···C23^vii^	3.5622
C7···H3	3.2681	H10···C24^x^	2.7110
C7···H7	3.4234	H10···H11A^xii^	3.5777
C8···H3	3.2732	H10···H12B^xii^	3.0917
C8···H5	3.2941	H10···H16A^vii^	2.8818
C8···H18A	3.3630	H10···H17B^vi^	3.5483
C9···H1	3.2388	H10···H18A^x^	2.7485
C9···H2	3.2679	H10···H19B^x^	2.4185
C9···H4	3.2564	H10···H20C^x^	2.5150
C9···H7	2.9583	H11A···O1^iv^	3.2773
C10···H1	2.4837	H11A···C4^iv^	3.0440
C10···H11A	2.7433	H11A···C12^xi^	3.5332
C10···H12B	2.5328	H11A···C18^xv^	3.3525
C14···H1	3.2488	H11A···C19^xv^	3.5303
C14···H5	2.9872	H11A···H2^iv^	2.4907
C15···H1	2.5425	H11A···H8^ii^	3.3950
C15···H5	2.4890	H11A···H9^xv^	3.2888
C15···H6	3.2376	H11A···H10^xv^	3.5777
C15···H7	2.6946	H11A···H14B^xi^	2.9576
C16···H5	3.4501	H11A···H15C^xi^	3.2071
C16···H9	3.2684	H11A···H18A^iv^	3.4247
C17···H10	3.2702	H12B···O4^v^	3.5621
C18···H7	3.2742	H12B···C18^xv^	3.5459
C19···H8	3.2649	H12B···C19^xv^	3.1146
C20···H1	3.2870	H12B···C21^xv^	3.4887
C20···H5	2.9435	H12B···C23^v^	2.9250
C20···H8	3.2797	H12B···H10^xv^	3.0917
C20···H10	3.2989	H12B···H16A^v^	2.4860
C21···H6	3.1519	H12B···H17B^v^	2.4932
C21···H7	3.3053	H12B···H18A^iv^	2.9489
C21···H9	3.2729	H12B···H20C^iv^	3.4795
C22···H6	2.5672	H13A···O1^i^	3.4433
C22···H16A	2.6191	H13A···O4^v^	2.8541
C22···H17B	2.6478	H13A···C17^iii^	3.3009
H1···H7	3.3618	H13A···C18^iii^	2.9809
H2···H3	2.3180	H13A···H4^xv^	3.5528
H3···H4	2.3455	H13A···H5^xv^	3.0760
H4···H5	2.3310	H13A···H8^iii^	3.2930
H5···H7	3.4322	H13A···H9^iii^	2.7331
H7···H8	2.3115	H13A···H16A^v^	3.1643
H8···H9	2.3664	H14B···O1^i^	2.8415
H9···H10	2.3060	H14B···C17^ii^	3.1314
H11A···H13A	2.8577	H14B···C18^ii^	3.1837
H11A···H14B	2.3845	H14B···H2^i^	3.2824
H11A···H15C	2.3470	H14B···H8^ii^	2.7242
H12B···H13A	2.3474	H14B···H9^iii^	3.2979
H12B···H14B	2.8576	H14B···H9^ii^	2.8269
H12B···H15C	2.3843	H14B···H11A^xi^	2.9576
H16A···H18A	2.8558	H14B···H14B^xi^	3.4237
H16A···H19B	2.3937	H14B···H15C^xi^	3.2436
H16A···H20C	2.3365	H15C···C16^xv^	2.9958
H17B···H18A	2.3357	H15C···C17^xv^	2.9258
H17B···H19B	2.8557	H15C···C18^xv^	3.1156
H17B···H20C	2.3946	H15C···C19^xv^	3.3449
O1···H4^ix^	3.3409	H15C···C20^xv^	3.2647
O1···H8^ii^	2.6034	H15C···C21^xv^	3.4265
O1···H9^iii^	2.4059	H15C···H2^i^	2.9882
O1···H11A^viii^	3.2773	H15C···H5^xv^	3.2508
O1···H13A^i^	3.4433	H15C···H7^xv^	3.4211
O1···H14B^i^	2.8415	H15C···H8^xv^	3.3256
O2···H16A^v^	2.9985	H15C···H11A^xi^	3.2071
O2···H17B^iv^	3.4220	H15C···H14B^xi^	3.2436
O2···H17B^v^	3.4154	H16A···O2^v^	2.9985
O2···H18A^iv^	2.5154	H16A···O3^v^	2.9902
O2···H20C^iv^	3.5896	H16A···C10^v^	2.9668
O2···H20C^v^	2.5977	H16A···C11^v^	3.0820
O3···H2^iv^	3.5373	H16A···C19^vii^	3.3374
O3···H8^ii^	2.6912	H16A···H9^vii^	3.5282
O3···H9^iii^	2.9268	H16A···H10^vii^	2.8818
O3···H16A^v^	2.9902	H16A···H12B^v^	2.4860
O4···H5^vi^	2.4848	H16A···H13A^v^	3.1643
O4···H6^vi^	3.5171	H16A···H19B^v^	3.0400
O4···H12B^v^	3.5621	H17B···O2^viii^	3.4220
O4···H13A^v^	2.8541	H17B···O2^v^	3.4154
O5···H10^vii^	3.4783	H17B···N2^vi^	2.8767
N2···H1^vii^	2.7418	H17B···C11^v^	3.4166
N2···H17B^vi^	2.8767	H17B···C13^vi^	3.4118
N2···H20C^x^	3.3870	H17B···C21^vi^	3.4823
C1···H4^iv^	3.5997	H17B···C24^xiii^	3.4851
C1···H6^vii^	3.5653	H17B···H6^vi^	3.5016
C2···H3^iv^	3.3558	H17B···H10^vi^	3.5483
C3···H4^ix^	3.5833	H17B···H12B^v^	2.4932
C3···H8^ii^	3.3404	H17B···H18A^xiii^	3.3506
C3···H9^iii^	3.2308	H17B···H20C^xiii^	2.7540
C4···H7^ix^	3.4854	H18A···O2^viii^	2.5154
C4···H11A^viii^	3.0440	H18A···C11^viii^	3.5891
C5···H7^ix^	3.1398	H18A···C19^iii^	3.5463
C6···H1^viii^	3.4832	H18A···H10^iii^	2.7485
C6···H2^ix^	3.3689	H18A···H11A^viii^	3.4247
C6···H6^vi^	3.3152	H18A···H12B^viii^	2.9489
C6···H7^viii^	3.3133	H18A···H17B^xiii^	3.3506
C7···H2^ix^	3.3674	H18A···H20C^xiii^	3.2218
C7···H6^vi^	3.0373	H19B···C19^iii^	3.3639
C8···H3^ix^	3.3698	H19B···C23^v^	3.5003
C9···H3^ix^	3.4764	H19B···C24^v^	3.1740
C10···H3^iv^	3.2903	H19B···H10^iii^	2.4185
C10···H8^ii^	3.5754	H19B···H16A^v^	3.0400
C10···H16A^v^	2.9668	H19B···H19B^v^	2.4826
C10···H20C^v^	3.2797	H19B···H20C^v^	3.2698
C11···H2^iv^	3.3478	H20C···O2^viii^	3.5896
C11···H8^ii^	3.3825	H20C···O2^v^	2.5977
C11···H16A^v^	3.0820	H20C···N2^iii^	3.3870
C11···H17B^v^	3.4166	H20C···C10^v^	3.2797
C11···H18A^iv^	3.5891	H20C···C19^iii^	3.3479
C12···H2^i^	3.5014	H20C···C23^xiii^	3.4856
C12···H8^ii^	3.4407	H20C···C24^xiii^	3.4241
C12···H9^iii^	3.3470	H20C···H1^v^	3.4058
C12···H11A^xi^	3.5332	H20C···H10^iii^	2.5150
C13···H1^vii^	3.0655	H20C···H12B^viii^	3.4795
C13···H5^vi^	3.5802	H20C···H17B^xiii^	2.7540
C13···H17B^vi^	3.4118	H20C···H18A^xiii^	3.2218
C15···H6^vii^	3.4641	H20C···H19B^v^	3.2698
C16···H2^ix^	3.5155	H20C···H20C^xiii^	3.0947
			
C10—O3—C11	116.03 (17)	O4—C22—C14	122.8 (2)
C22—O5—C23	116.67 (17)	O5—C22—C14	113.12 (18)
C1—N1—C9	120.57 (19)	O5—C23—C24	106.50 (18)
C1—N1—C15	119.36 (17)	N1—C1—H1	118.012
C9—N1—C15	120.07 (15)	C2—C1—H1	118.004
C13—N2—C21	117.3 (2)	C5—C4—H2	119.617
N1—C1—C2	123.98 (18)	C8—C4—H2	119.625
C1—C2—C3	120.06 (16)	C4—C5—H3	119.978
C1—C2—C10	114.17 (18)	C6—C5—H3	119.971
C3—C2—C10	125.77 (19)	C5—C6—H4	119.697
O1—C3—C2	125.66 (17)	C7—C6—H4	119.692
O1—C3—C8	119.87 (18)	C6—C7—H5	120.452
C2—C3—C8	114.47 (19)	C9—C7—H5	120.455
C5—C4—C8	120.8 (2)	O3—C11—H11A	110.326
C4—C5—C6	120.1 (2)	O3—C11—H12B	110.317
C5—C6—C7	120.61 (18)	C12—C11—H11A	110.316
C6—C7—C9	119.1 (2)	C12—C11—H12B	110.320
C3—C8—C4	119.6 (2)	H11A—C11—H12B	108.569
C3—C8—C9	122.01 (18)	C11—C12—H13A	109.471
C4—C8—C9	118.38 (17)	C11—C12—H14B	109.471
N1—C9—C7	120.1 (2)	C11—C12—H15C	109.470
N1—C9—C8	118.82 (16)	H13A—C12—H14B	109.471
C7—C9—C8	121.09 (19)	H13A—C12—H15C	109.466
O2—C10—O3	123.53 (17)	H14B—C12—H15C	109.479
O2—C10—C2	123.3 (2)	N2—C13—H6	117.274
O3—C10—C2	113.22 (18)	C14—C13—H6	117.275
O3—C11—C12	106.99 (18)	C17—C16—H7	119.663
N2—C13—C14	125.5 (2)	C20—C16—H7	119.668
C13—C14—C15	116.96 (19)	C16—C17—H8	119.901
C13—C14—C22	116.01 (18)	C18—C17—H8	119.897
C15—C14—C22	127.0 (2)	C17—C18—H9	119.931
N1—C15—C14	122.56 (18)	C19—C18—H9	119.919
N1—C15—C20	117.19 (18)	C18—C19—H10	119.481
C14—C15—C20	120.3 (2)	C21—C19—H10	119.469
C17—C16—C20	120.7 (3)	O5—C23—H16A	110.426
C16—C17—C18	120.2 (2)	O5—C23—H17B	110.425
C17—C18—C19	120.1 (2)	C24—C23—H16A	110.425
C18—C19—C21	121.1 (3)	C24—C23—H17B	110.428
C15—C20—C16	123.4 (2)	H16A—C23—H17B	108.636
C15—C20—C21	117.64 (18)	C23—C24—H18A	109.469
C16—C20—C21	118.91 (18)	C23—C24—H19B	109.467
N2—C21—C19	118.7 (2)	C23—C24—H20C	109.462
N2—C21—C20	122.31 (18)	H18A—C24—H19B	109.476
C19—C21—C20	119.01 (19)	H18A—C24—H20C	109.473
O4—C22—O5	124.09 (19)	H19B—C24—H20C	109.480
			
C10—O3—C11—C12	−163.33 (14)	C3—C8—C9—N1	1.7 (3)
C10—O3—C11—H11A	76.6	C3—C8—C9—C7	−177.41 (14)
C10—O3—C11—H12B	−43.3	C4—C8—C9—N1	−179.99 (15)
C11—O3—C10—O2	1.9 (3)	C4—C8—C9—C7	0.9 (3)
C11—O3—C10—C2	−178.65 (13)	O3—C11—C12—H13A	63.4
C22—O5—C23—C24	177.74 (14)	O3—C11—C12—H14B	−56.6
C22—O5—C23—H16A	57.8	O3—C11—C12—H15C	−176.6
C22—O5—C23—H17B	−62.3	H11A—C11—C12—H13A	−176.6
C23—O5—C22—O4	−0.3 (3)	H11A—C11—C12—H14B	63.4
C23—O5—C22—C14	179.11 (13)	H11A—C11—C12—H15C	−56.6
C1—N1—C9—C7	−179.62 (14)	H12B—C11—C12—H13A	−56.6
C1—N1—C9—C8	1.3 (3)	H12B—C11—C12—H14B	−176.6
C9—N1—C1—C2	−2.6 (3)	H12B—C11—C12—H15C	63.4
C9—N1—C1—H1	177.4	N2—C13—C14—C15	−0.1 (3)
C1—N1—C15—C14	88.7 (2)	N2—C13—C14—C22	179.21 (15)
C1—N1—C15—C20	−92.14 (18)	H6—C13—C14—C15	179.9
C15—N1—C1—C2	178.02 (14)	H6—C13—C14—C22	−0.8
C15—N1—C1—H1	−2.0	C13—C14—C15—N1	−179.07 (14)
C9—N1—C15—C14	−90.68 (19)	C13—C14—C15—C20	1.8 (3)
C9—N1—C15—C20	88.5 (2)	C13—C14—C22—O4	−6.1 (3)
C15—N1—C9—C7	−0.2 (3)	C13—C14—C22—O5	174.47 (14)
C15—N1—C9—C8	−179.35 (14)	C15—C14—C22—O4	173.10 (16)
C13—N2—C21—C19	−179.53 (15)	C15—C14—C22—O5	−6.3 (3)
C13—N2—C21—C20	0.3 (3)	C22—C14—C15—N1	1.7 (3)
C21—N2—C13—C14	−0.9 (3)	C22—C14—C15—C20	−177.43 (15)
C21—N2—C13—H6	179.0	N1—C15—C20—C16	−1.1 (3)
N1—C1—C2—C3	0.8 (3)	N1—C15—C20—C21	178.46 (13)
N1—C1—C2—C10	−178.65 (15)	C14—C15—C20—C16	178.09 (15)
H1—C1—C2—C3	−179.2	C14—C15—C20—C21	−2.4 (3)
H1—C1—C2—C10	1.3	C17—C16—C20—C15	178.37 (15)
C1—C2—C3—O1	−178.32 (16)	C17—C16—C20—C21	−1.2 (3)
C1—C2—C3—C8	2.0 (3)	C20—C16—C17—C18	0.7 (3)
C1—C2—C10—O2	−4.8 (3)	C20—C16—C17—H8	−179.3
C1—C2—C10—O3	175.71 (15)	H7—C16—C17—C18	−179.3
C3—C2—C10—O2	175.77 (16)	H7—C16—C17—H8	0.7
C3—C2—C10—O3	−3.7 (3)	H7—C16—C20—C15	−1.6
C10—C2—C3—O1	1.1 (3)	H7—C16—C20—C21	178.8
C10—C2—C3—C8	−178.61 (15)	C16—C17—C18—C19	0.3 (3)
O1—C3—C8—C4	−1.2 (3)	C16—C17—C18—H9	−179.7
O1—C3—C8—C9	177.06 (15)	H8—C17—C18—C19	−179.7
C2—C3—C8—C4	178.49 (14)	H8—C17—C18—H9	0.3
C2—C3—C8—C9	−3.2 (3)	C17—C18—C19—C21	−0.7 (3)
C5—C4—C8—C3	178.51 (15)	C17—C18—C19—H10	179.3
C5—C4—C8—C9	0.2 (3)	H9—C18—C19—C21	179.2
C8—C4—C5—C6	−1.0 (3)	H9—C18—C19—H10	−0.8
C8—C4—C5—H3	178.9	C18—C19—C21—N2	−179.92 (16)
H2—C4—C5—C6	179.0	C18—C19—C21—C20	0.2 (3)
H2—C4—C5—H3	−1.0	H10—C19—C21—N2	0.1
H2—C4—C8—C3	−1.5	H10—C19—C21—C20	−179.8
H2—C4—C8—C9	−179.8	C15—C20—C21—N2	1.3 (3)
C4—C5—C6—C7	0.9 (3)	C15—C20—C21—C19	−178.85 (14)
C4—C5—C6—H4	−179.1	C16—C20—C21—N2	−179.13 (14)
H3—C5—C6—C7	−179.1	C16—C20—C21—C19	0.7 (3)
H3—C5—C6—H4	0.9	O5—C23—C24—H18A	65.3
C5—C6—C7—C9	0.1 (3)	O5—C23—C24—H19B	−54.7
C5—C6—C7—H5	−179.9	O5—C23—C24—H20C	−174.7
H4—C6—C7—C9	−179.9	H16A—C23—C24—H18A	−174.8
H4—C6—C7—H5	0.1	H16A—C23—C24—H19B	65.2
C6—C7—C9—N1	179.86 (15)	H16A—C23—C24—H20C	−54.8
C6—C7—C9—C8	−1.0 (3)	H17B—C23—C24—H18A	−54.6
H5—C7—C9—N1	−0.1	H17B—C23—C24—H19B	−174.6
H5—C7—C9—C8	179.0	H17B—C23—C24—H20C	65.4

Symmetry codes: (i) −*x*+1, −*y*+1, −*z*; (ii) −*x*+1, −*y*, −*z*; (iii) *x*, *y*+1, *z*; (iv) *x*+1, *y*, *z*; (v) −*x*+1, −*y*+1, −*z*+1; (vi) −*x*, −*y*, −*z*+1; (vii) −*x*+1, −*y*, −*z*+1; (viii) *x*−1, *y*, *z*; (ix) −*x*, −*y*, −*z*; (x) *x*, *y*−1, *z*; (xi) −*x*+2, −*y*+1, −*z*; (xii) *x*−1, *y*−1, *z*; (xiii) −*x*, −*y*+1, −*z*+1; (xiv) −*x*−1, −*y*, −*z*; (xv) *x*+1, *y*+1, *z*.
